# Nuclear Receptor Subfamily 4 Group A Member 1 (NR4A1) Promotes the Adipogenesis of Intramuscular Preadipocytes through PI3K/AKT Pathway in Goats

**DOI:** 10.3390/ani14142051

**Published:** 2024-07-12

**Authors:** Jiani Xing, Jianying Zheng, Sheng Cui, Jinling Wang, Yong Wang, Yanyan Li, Jiangjiang Zhu, Yaqiu Lin

**Affiliations:** 1 Key Laboratory of Qinghai-Tibetan Plateau Animal Genetic Resource Reservation and Utilization, Ministry of Education, Southwest Minzu University, Chengdu 610041, China; 80300244@swun.edu.cn (J.X.); 17882083378@163.com (J.Z.); 13072803853@163.com (S.C.); liyanyan@swun.edu.cn (Y.L.); zhujiang4656@swun.edu.cn (J.Z.); 2 College of Animal Husbandry and Veterinary Medicine, Southwest Minzu University, Chengdu 610041, China; 3 College of Life Science and Biotechnology, Mianyang Teachers’ College, Mianyang 621000, China; wjl08309625@163.com

**Keywords:** goat, *NR*4*A*1, intramuscular preadipocyte, adipogenesis

## Abstract

**Simple Summary:**

Nuclear Receptor Subfamily 4 Group A Member 1 (NR4A1) acts as a transcription factor to participate in lots of physiological activities including cell proliferation and cell differentiation. In this study, we found that NR4A1 promoted goat intramuscular preadipocyte differentiation through the PI3K/AKT pathway. This study provides important information about NR4A1 in the intramuscular preadipocyte differentiation in goats and therefore identifies a target for goat meat quality improvement studies.

**Abstract:**

As a transcription factor, Nuclear Receptor Subfamily 4 Group A Member 1 (NR4A1) binds to downstream target genes to participate in cell proliferation and cell differentiation. We found that the NR4A1 reached the highest expression at 60 h after the differentiation of goat intramuscular preadipocytes. Overexpression of goat NR4A1 increased the number of intracellular lipid droplets and up-regulated the expression of adipocyte-differentiation-related marker genes including *AP2*, *SREBP1*, *ACC*, *GPAM*, and *DGAT2*, while the relative expression levels of *Pref-1* and *HSL* were significantly decreased. On the contrary, after NR4A1 was knocked down by siRNA, the number of intracellular lipid droplets and the relative expression levels of *LPL*, *CEBPα*, *CEBPβ*, *ACC*, and *DGAT2* were significantly decreased, and the relative expression levels of *Pref-1* and *HSL* were significantly up-regulated. These results suggest that NR4A1 promotes the differentiation of goat intramuscular preadipocytes. Transcriptome sequencing was carried out after overexpression of goat NR4A1, and the KEGG enrichment analysis result showed that the most differentially expressed genes were related to adipocyte differentiation and were enriched in the PI3K-Akt signaling pathway. LY249002, an inhibitor of the PI3K-Akt signaling pathway, was introduced and decreased the number of intracellular lipid droplets, and the relative expression levels of *C/EBPα*, *SREBP1*, *AP2*, *C/EBPβ*, *GPAM*, *ACC*, *DGAT1*, *DGAT2*, and *ATGL* were decreased accordingly. The above results indicate that overexpression of goat NR4A1 may promote the differentiation of intramuscular preadipocytes through the PI3K-Akt signaling pathway.

## 1. Introduction

The goat breeding industry plays a vital role in China’s animal husbandry, and goat meat is favored by lots of consumers owing to its high protein content, low content of fat and cholesterol, and rich, abundant essential amino acids. Therefore, the improvement of the quality of mutton becomes an urgent problem to be solved. Intramuscular fat (IMF) content is one of the key determinant factors that affects the flavor, tenderness, and juiciness of meat and is determined by preadipocyte differentiation and lipid metabolism. The differentiation of preadipocytes is a complex process with changes of cell morphology, structure, and function, in which functional genes and transcription factors play a major role. Thus, there is great significance to revealing the key candidate genes and transcription factors and the regulatory mechanism of intramuscular fat deposition in goats.

NR4A1 (also known as Nur77 and TR3) is a member of the orphan nuclear receptor transcription factor family and one of the first early response genes to be discovered by rapid activation of NGF in PC12 pheochromocytoma cells [1]. NR4A1 is expressed in a variety of tissues and participates in a variety of physiological activities, including cell proliferation and apoptosis [2,3], and can be induced by physical stimulation and physiological signal expression [4].

In recent years, more and more studies have proved that NR4A1 is involved in the regulation of animal fat metabolism [5], for instance, NR4A1 knockout mice, compared with wild-type mice, fed with high-fat diet were more prone to obesity [6]. However, there are two completely opposite theories about the role of NR4A1 in adipocyte differentiation. Chao et al. [7] also found that overexpression of NR4A1 can inhibit the differentiation of 3T3-F442A preadipocytes. However, Yi et al. [8] pointed out that the low expression of NR4A1 can reduce the body weight, blood lipid, and blood sugar of type 2 diabetic mice, and the size of adipocytes and the volume of lipid droplets decreased accordingly. Jung et al. [9] found that NR4A1 knockout inhibits adipogenesis and differentiation of 3T3-L1 preadipocytes, which is accompanied by a decrease in mitotic clone amplification (MCE) and the MCE-required and cell-cycle-related gene expression (including expression of cyclin An and cyclin D1) in the early stage of adipogenesis. The latest studies have shown that NR4A1 can directly inhibit the transcription of PPARγ by activating the expression of GATA2, and indirectly up-regulate p53 to reduce the expression of SREBP1c and downstream gene *FAS*, thus inhibiting adipogenesis [2], but it has also been reported that overexpression of NR4A1 or interference with NR4A1 in 3T3-L1 cells can reduce adipocyte differentiation, while transient overexpression can promote lipid accumulation [3]. The above studies suggest that NR4A1 has an effect on adipocyte differentiation and lipid metabolism, but there are differences in the mode of action, and the mechanism has not been fully explained. At the same time, the effect of NR4A1 on the differentiation of ruminant preadipocytes and its possible mechanism have not been reported yet.

Thus, the cultured goat intramuscular preadipocytes were introduced to reveal the function and the molecular mechanism of *NR4A1* in regulating IMF deposition. The expression trend of goat *NR4A1* in intramuscular adipocytes at different differentiation stages was detected by qPCR; overexpression and RNA interference were introduced to explicate the effect of goat *NR4A1* on intramuscular adipocyte differentiation; the number of lipid droplets of intramuscular adipocytes was observed by oil red O staining; and the relative expression levels of adipose differentiation marker genes and lipid-metabolism-related genes were detected by qPCR. RNA-Seq, qPCR, and bioinformatics analysis were used to further explore the regulatory mechanism of goat NR4A1 on adipogenesis and lipid metabolism.

## 2. Materials and Methods

### 2.1. Animals and Cell Culture

The animal experimentation study received approval from the Laboratory Animal Ethics Committee at Southwest Minzu University and the Animal Disease Control Center in Sichuan Province, China. The Jianzhou Daer goats (*Capra hircus*) (N 3) were purchased from Sichuan Tiandi Goat Biological Engineering Co., Ltd. (Chengdu, China). Goat intramuscular preadipocytes were isolated and cultured in accordance with previously described methods [10,11]. Concisely, Longissimus dorsi muscle samples were collected from 7-day-old Jianzhou Daer goats and then sheared. Intramuscular preadipocytes were isolated by collagenase type II digestion (Gbico, Thermo, Waltham, MA, USA) containing 2 mg/mL. Finally, preadipocytes were cultured in DMEM/F12 growth medium with 10% FBS and 1% P/S.

### 2.2. Induction of Goat Intramuscular Preadipocyte

The goat intramuscular preadipocytes were cultured in DMEM/F12 medium containing 10% fetal bovine serum. When the preadipocyte cells’ confluency reached 80%, the differentiation medium (DMEM/F12 medium with 10% FBS and 100 μm/L oleic acid (Sigma)) was added.

### 2.3. Construction of NR4A1 Overexpression Vector

The primers listed in the following were designed according to the sequence of goat NR4A1 (MN197544.1), and the sequences were as follows:

sense primer: 5′-CGGGGTACCATGCCCTGTATCCAAGCCC-3′;

antisense primer: 5′-ATAAGAATGCGGCCGCTCAGAAGGGCAGTGTGTCC-3′.

The previously constructed NR4A1-PMD19T plasmid was used as the template, the primers containing restriction enzyme sites were used for amplification, and then gel extraction was carried out. The gel extraction product and the pcDNA3.1(+) vector were digested with *Kpn*Ⅰ and *Not*Ⅰ at 37 °C for 1 h, respectively, followed by purification, and then ligated by T4 ligase at 16 °C for 10–14 h. The ligated products were all transformed into DH5α competent cells and spread on LB solid medium containing Amp and cultured at 37 °C for overnight. The next day, monoclonals were randomly selected for colony PCR testing, and the positive monoclonals were transferred in liquid LB medium containing Amp for overnight culture and plasmid extraction. The plasmid concentration was detected by Nanodrop (TY20190063.IMPLEN, Munich, Germany) and then stored at −20 °C.

### 2.4. Synthesis of siRNA

Two pairs of siRNAs were designed and synthesized by Invitrogen (Carlsbad, CA, USA), named NR4A1 siRNA-1 (5′-CAUGGUGAAGGAAGUUGUCCGGACA-3′, 5′-UGUCCGGACAACUUCCUUCACCAUG-3′) and NR4A1 siRNA-2 (5′-GAGUCCGCCUUUCUGGAGCUCUUUA-3′, 5′-UAAAGAGCUCCAGAAAGGCGGACUC-3′). Negative control was provided by Invitrogen, and the sequences were sense primer 5′-UUCUCCGAACGUGUCACGUTT-3′ and antisense primer 5′-ACGUGACACGUUCGGAGAATT-3′.

### 2.5. Cell Transfection

When the cells were passaged to the third generation, and the intramuscular adipocyte cells were fused to 80%, the cell samples were collected after 48 h of differentiation induced by the proper amount of overexpression vector, NR4A1 siRNA-1, and NR4A1 siRNA-2. During inhibitor transfection, the experimental group first transfected goat intramuscular preadipocytes with pcDNA3.1-NR4A1, and then added the appropriate amount of LY294002 inhibitor 6 h later. Each processing setting has 3 duplicates.

### 2.6. RNA Extraction and Quantitative Real-Time Polymerase Chain Reaction (qPCR)

Total RNA was extracted from cultured cell samples using Trizol reagent (TaKaRa, Kusatsu, Shiga, Japan). The integrity of the total RNA was detected by 2% agarose gel electrophoresis, and the concentration was determined using ultraviolet spectrophotometer. For each cell sample, 1 µg of total RNA was reverse transcribed by Revert Aid First Strand cDNA Synthesis Kit (Thermo) according to the manufacturer’s instructions. Ubiquitously expressed prefoldin-like chaperone (*UXT*) was selected to normalize the expression levels (geometric mean was used to calculate the internal control). The primer information for qPCR is listed in Table 1. SYBR^®^ Premix Ex Taq TM (2×) (Takara) and CFX96 (Bio-Rad) were used to perform qPCR. The qPCR reaction steps were as follows: 95 °C 30 s, 95 °C 10 s, 60 °C 30 s, and 72 °C 30 s, 39 cycles in total. The 2^−ΔΔ^Ct method was used to analyze the expression of each gene.

### 2.7. Oil Red O Staining

Intramuscular adipocytes were cultured in 24-well plates and visualized by oil red O staining. Intramuscular adipocytes were fixed with 500 µL of 10% formaldehyde solution for 30 min after being washed twice with PBS, and then the fixed adipocytes were rinsed twice in PBS and stained with 500 µL of oil red O working solution for 10 min. Subsequently, adipocytes were washed twice using PBS and observed or photographed under microscope. Finally, the lipids were extracted by 1 mL isopropyl alcohol, and the extracted solution was detected in 96-well plates by colorimeter at OD 490 nm.

### 2.8. Total RNA Extraction and RNA-Seq

Total RNA was extracted from the cells transfected with the NR4A1 overexpression vector (N = 3) and control vector (N = 3) using RNAiso Plus (TaKaRa, Kusatsu, Shiga, Japan) according to the manufacturer’s instructions (Invitrogen), and genomic DNA was removed using DNase I (TaKaRa, Kusatsu, Shiga, Japan). Then, RNA quality was determined by 2100 Bioanalyser (Agilen, Santa Clara, CA, USA) and quantified using the Nanodrop (TY20190063.IMPLEN, Munich, Germany). The RNA-Seq transcriptome library was prepared using the TruSeq TMRNA sample preparation Kit from Illumina (San Diego, CA, USA) using 1 μg of total RNA. Shortly, messenger RNA was isolated according to polyA selection by oligo(dT) beads and then fragmented by fragmentation buffer firstly. Secondly, double-stranded cDNA was synthesized using a SuperScript double-stranded cDNA synthesis kit (Invitrogen, Carlsbad, CA, USA) with random hexamer primers (Illumina). Then, the synthesized cDNA was subjected to end repair, phosphorylation, and ‘A’ base addition according to Illumina’s library construction protocol. Libraries were size-selected for cDNA target fragments of 300 bp on 2% Low-Range Ultra Agarose then were PCR amplified using Phusion DNA polymerase (NEB) for 15 PCR cycles. After being quantified by TBS380, the paired-end RNA-Seq sequencing library was sequenced with the Illumina HiSeq xten/NovaSeq 6000 sequencer (2 × 150 bp read length).

The differentially expressed mRNAs were randomly selected to verify the accuracy of the RNA-Seq results. The primer sequences are listed in Table 2.

### 2.9. Differentially Expressed Genes and KEGG Analysis

Cuffdiff provides statistical routines for determining differential expression in digital transcript or gene expression data using a model based on the negative binomial distribution [12]. Transcripts with a *p*-adjust < 0.05 were assigned as differentially expressed. KEGG is a database resource for understanding high-level functions and utilities of the biological system [13]. KOBAS software (3.0) was introduced to test the statistical enrichment of differentially expressed genes (DEGs) in KEGG pathways [14].

### 2.10. Data Analysis

All the data are provided as “Means ± SD”. One-way ANOVA was performed with SPSS software (26.0) to compare significance, followed by Duncan’s multiple comparison test. A *p* value less than 0.05 was considered a significant difference. All experiments were repeated three times.

## 3. Results

### 3.1. The Expression Patterns of NR4A1 Gene during Differentiation of Goat Intramuscular Adipocytes

**Figure 1:**
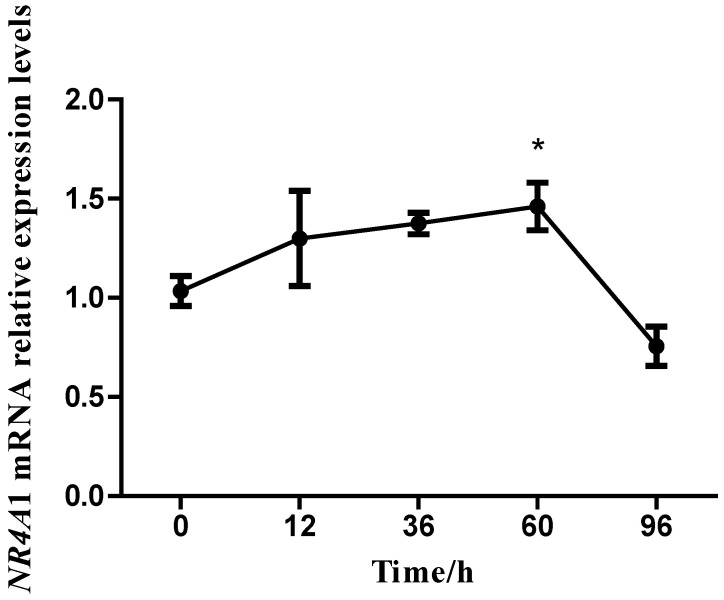
**Relative expression level of *NR4A1* during intramuscular adipocyte differentiation.** The expression level of NR4A1 mRNA was detected by qPCR, and the *UXT* gene was used as the reference gene. Data are expressed as “Means ± SD”, *n* = 6, * *p* < 0.05.

The regulatory effect of NR4A1 on goat intramuscular adipocyte differentiation has not been reported; thus, the expression pattern of *NR4A1* at different stages of intramuscular adipocyte differentiation was investigated first. The relative expression of *NR4A1* was detected at 0, 12, 36, 60, 96 h after differentiation induced by oleic acid. The result revealed that *NR4A1* expression showed an overall upward trend in the first 60 h, reached the peak at the 60 h, and then decreased after 60 h during induction. Taken together, *NR4A1* was considered to participate in the process of intramuscular preadipocyte differentiation in goats (Figure 1).

### 3.2. NR4A1 Overexpression Enhances Intramuscular Preadipocyte Differentiation

**Figure 2:**
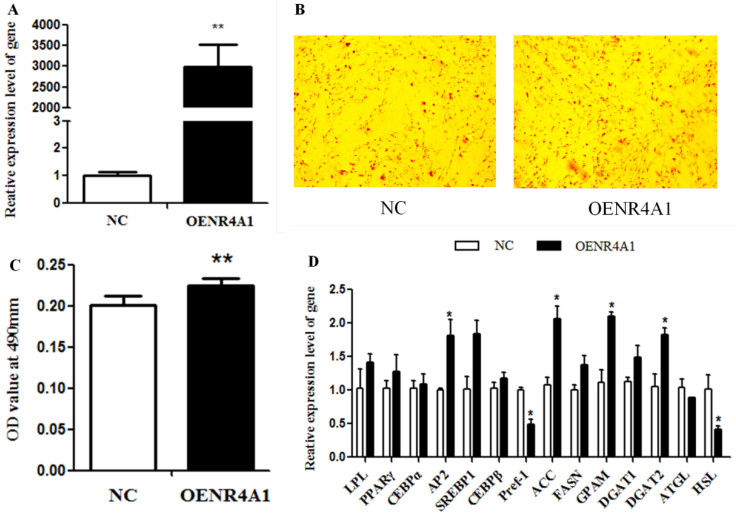
**Overexpression of *NR4A1* promotes the differentiation of intramuscular adipocytes in goats.** (**A**) *NR4A1* overexpression efficiency detection. (**B**) The result of oil red O staining images of 2-day induced differentiation between NC and NR4A1 overexpression groups. (**C**) The result of OD value at 490 nm. (**D**) The relative expression levels of goat intramuscular adipocyte differentiation marker genes and lipid-metabolism-related genes after overexpression of *NR4A1*. Data are expressed as “Means ± SD”, *n* = 3. ** *p* < 0.01, * *p* < 0.05.

In order to further investigate the function of NR4A1 in the adipogenesis of goat intramuscular adipocytes, NR4A1 was overexpressed in goat intramuscular preadipocytes using pcDNA3.1-NR4A1. After pcDNA3.1-NR4A1 was transfected into goat intramuscular adipocytes and differentiation induced for 48 h, the expression level of *NR4A1* was 2980.74 times higher than in the NC group (*p* < 0.01) (Figure 2A). The result of the oil red O staining indicated that the number of lipid droplets significantly increased after overexpression of *NR4A1* (Figure 2B). The semi-quantitative result also confirmed that NR4A1 could promote lipid droplet accumulation in goat intramuscular preadipocytes (*p* < 0.01) (Figure 2C). In addition, adipogenic and lipid metabolism genes were assessed by qPCR, and the results showed that overexpression of *NR4A1* up-regulated the expression of *AP2* (*p* < 0.05), *SREBP1* (*p* < 0.05), *ACC* (*p* < 0.05), *GPAM* (*p* < 0.05), and *DGAT2* (*p* < 0.05), and down-regulated the expression of *Pref-1* (*p* < 0.05) and *HSL* (*p* < 0.05). However, *LPL*, *PPARγ*, *C/EBPα*, *C/EBPβ*, *FASN*, *DGAT1*, and *ATGL* had no significant change after *NR4A1* overexpression (Figure 2D). Taken together, NR4A1 promoted the differentiation of goat intramuscular adipocytes.

### 3.3. NR4A1 Knockdown Inhibits Goat Intramuscular Adipocyte Differentiation

**Figure 3:**
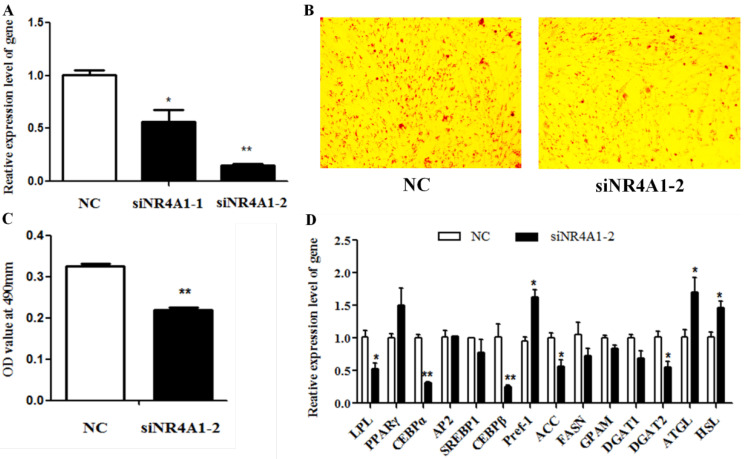
**Knockdown of *NR4A1* expression blocks the differentiation of intramuscular adipocytes in goats.** (**A**) The knockdown efficiency of *NR4A1*. (**B**) The result of oil red O staining images of 2-day induced differentiation between NC and NR4A1 overexpression groups. (**C**) The result of OD value at 490 nm. (**D**) The relative expression levels of goat intramuscular adipocyte differentiation marker genes and lipid-metabolism-related genes after knockdown of *NR4A1*. Data are expressed as “Means ± SD”, *n* = 3. ** *p* < 0.01, * *p* < 0.05.

To confirm the role of *NR4A1* in intramuscular adipogenic differentiation, the effect of *NR4A1* knockdown on intramuscular adipocyte differentiation was observed using two specific siRNAs (siNR4A1-1, siNR4A1-2). The relative mRNA expression of *NR4A1* was decreased ~44% by siNR4A1-1 (*p* < 0.01) and ~85% by siNR4A1-2 (*p* < 0.01) (Figure 3A). Thus, siNR4A1-2 was used for subsequent experiments. As expected, *NR4A1* knockdown blocked lipid droplet accumulation, based on the oil red O staining result (Figure 3B), and the quantitative results obtained by the OD value at 490 nm were consistent with the staining result (Figure 3C). At the gene expression level, *LPL*, *C/EBPα*, *C/EBPβ*, *ACC,* and *DGAT2* expression was significantly decreased (*p* < 0.05), and *Pref-1* and *HSL* expression was significantly increased (*p* < 0.05). Nevertheless, the expression of *PPARγ*, *AP2*, *SREBP1*, *FASN*, *GPAM*, and *DGAT1* was correspondingly unaffected (Figure 3D). Thus, knockdown of NR4A1 expression inhibited the adipogenesis of goat intramuscular preadipocytes.

### 3.4. KEGG Enrichment Analysis

**Figure 4:**
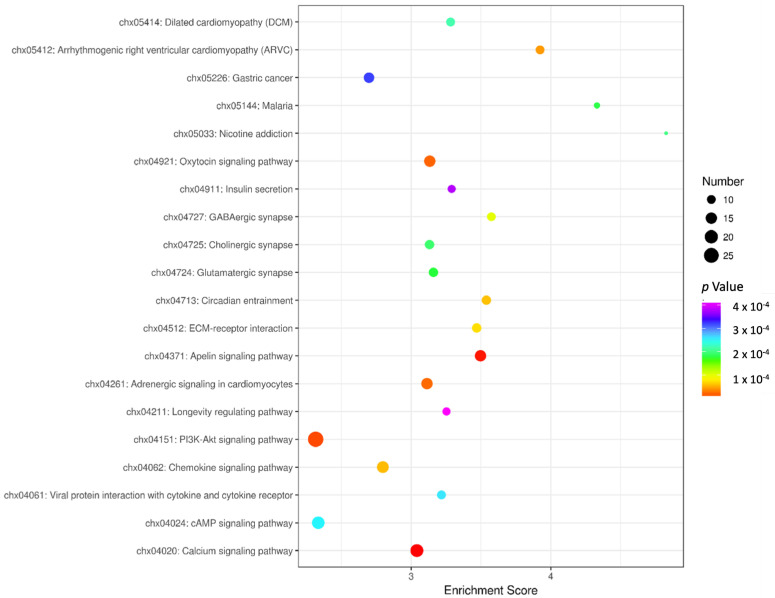
KEGG enrichment analysis of differentially expressed mRNAs.

There were 690 differentially expressed genes (DEGs) in total with NR4A1 overexpression compared with in the NC group, in which 446 genes were up-regulated and 244 genes were down-regulated. KEGG enrichment analysis showed that the DEGs were significantly enriched in 86 pathways. The most significant enrichment was in the Calcium signaling pathway, Apelin signaling pathway, PI3K-Akt signaling pathway, Oxytocin signaling pathway, Adrenergic signaling in cardiomyocytes, Arrhythmogenic right ventricular cardiomyopathy, Chemokine signaling pathway, Circadian entrainment, ECM–receptor interaction, and GABAergic synapse (Figure 4). In addition, the Camp signaling pathway, IL-17 signaling pathway, cGMP-PKG signaling pathway, TNF signaling pathway, FoxO signaling pathway, Wnt signaling pathway, Hippo signaling pathway, AMPK signaling pathway, p53 signaling pathway, PPAR signaling pathway, and other pathways related to adipose differentiation were significantly enriched.

**Figure 5:**
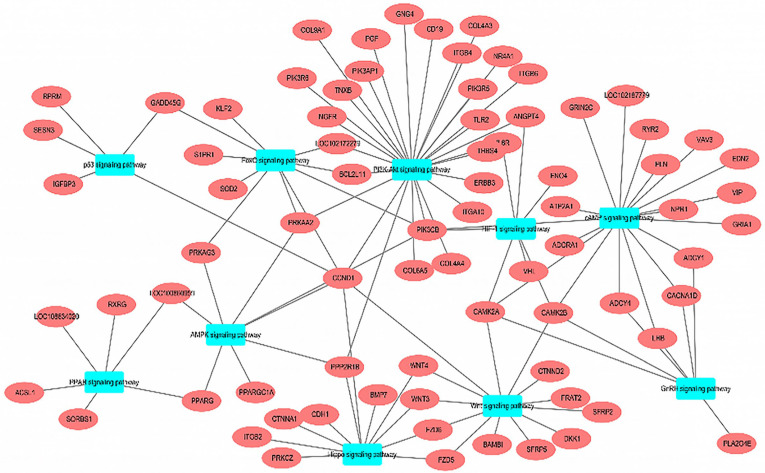
Differentially expressed mRNAs were enriched into adipocyte differentiation and lipid metabolism pathways.

The PI3K-Akt signaling pathway, Camp signaling pathway, PPAR signaling pathway, AMPK signaling pathway, and Wnt signaling pathway were selected to analyze the signaling pathways related to adipocyte differentiation and lipid metabolism (Figure 5). It was found that there were different degrees of differentially expressed gene enrichment in these pathways, with the largest number of genes enriched in the PI3K-Akt signaling pathway, and the second pathway with the most gene enrichment, such as enrichment of P*IK3CB*, *COL6A5*, *ITGB6*, and *PRKAA2*, was the cAMP signaling pathway. Differentially expressed genes such as *ITGA10*, *IL6R*, and *NR4A1* were enriched in the PI3K-Akt signaling pathway, while *PIK3CB*, *EDN2*, *VAV3*, *NPR1*, *CAMK2B*, *ADCY1*, and *GRIA1* were enriched in the cAMP signaling pathway.

The most differentially expressed genes and the *NR4A1* gene were all enriched in the PI3K-Akt signaling pathway, and the PI3K-Akt signaling pathway was chosen for further research.

### 3.5. RNA-Seq Verification Result

**Figure 6:**
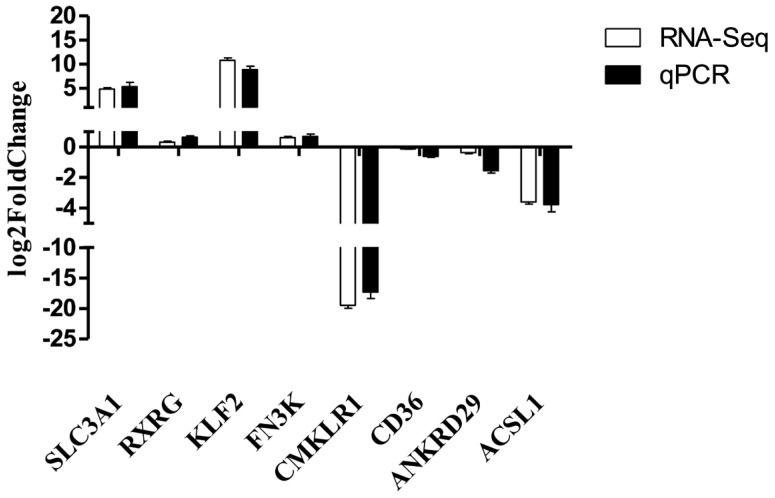
Verification of differentially expressed mRNAs.

In this study, eight differentially expressed mRNAs were randomly selected for expression detection by qPCR. The results confirmed (Figure 6) the RNA-Seq data, indicating that the RNA-Seq data in this study are highly reliable.

### 3.6. LY294002 Inhibits Overexpression of NR4A1 and Promotes Differentiation of Goat Intramuscular Adipocytes

**Figure 7:**
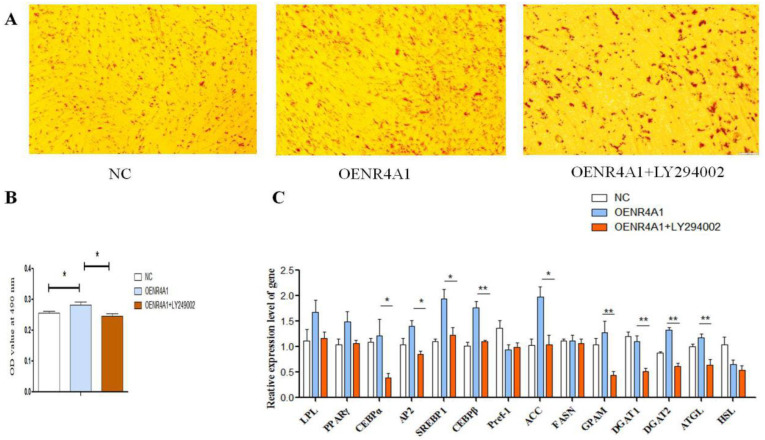
**LY249002 blocks *NR4A1* overexpression and promotes the differentiation of intramuscular preadipocytes in goats.** (**A**) The resulting oil red O staining images of 2-day induced differentiation among NC, NR4A1 overexpression, and NR4A1 overexpression with LY294002 added groups. (**B**) The result of OD value at 490 nm. (**C**) Overexpression of *NR4A1* followed by addition of LY249002 to the relative expression levels of goat intramuscular adipocyte differentiation marker genes and lipid-metabolism-related genes. Data are expressed as “Means ± SD”, *n* = 3. ** *p* < 0.01, * *p* < 0.05.

After overexpression of NR4A1 in goat intramuscular adipocytes, LY249002 (10 μM), an inhibitor of the PI3K-Akt signaling pathway, was added so the morphological changes of lipid droplet accumulation in goat intramuscular adipocytes could be observed to clarify whether NR4A1 plays a certain regulatory role in goat intramuscular adipocyte differentiation through the PI3K-Akt signaling pathway. The results of oil red O staining showed that the number of lipid droplets increased by NR4A1 overexpression was significantly decreased by the inhibitor of LY249002 (Figure 7A). The semi-quantitative results were consistent with the results mentioned earlier, as indicated by the OD value (Figure 7B). The expression level of genes related to adipocyte differentiation, such as *C/EBP α*, *AP2*, *SREBP1*, and *C/EBP β*, and genes involved in lipid metabolism, such as *ACC*, *GPAM*, *DGAT1*, *DGAT2*, and *ATGL*, was also decreased significantly *(p* < 0.05) (Figure 7C).

## 4. Discussion

Intramuscular fat deposition is determined by the increase in the preadipocyte cell number and the preadipocyte differentiation, which are precisely regulated by various key genes and transcription factors. Therefore, revealing the crucial transcription factors that influence adipogenesis to enhance intramuscular fat deposition is of great significance. The NR4A family participates in a variety of physiological processes in mammals, such as cell proliferation and differentiation, and NR4A1 is one of the family members [2,3]. Based on the results for NR4A1 in 3T3-L1 and 3T3-F442A cell lines, it was confirmed that NR4A1 is involved in adipocyte differentiation, but the mechanism remains unclear. To clarify the effect of goat NR4A1 on the differentiation of intramuscular preadipocytes and the underlying mechanism, the expression pattern of NR4A1 in different differentiation stages of goat intramuscular preadipocytes was analyzed, and it was found that goat NR4A1 showed an upward trend in the first 60 h of differentiation, and a downward trend during 60 h to 96 h, which showed a significant difference in the expression of NR4A1 before and after intramuscular preadipocyte differentiation, indicating that NR4A1 may promote the differentiation of intramuscular preadipocytes in the early stage. Fumoto et al. pointed out that NR4A1 was induced and expressed in the early stage of 3T3-L1 differentiation [3], but Veum et al. proposed that NR4A1 was down-regulated during the differentiation of primary human preadipocytes [15]. This difference may be caused by species specificity and different sampling sites; therefore, the role of NR4A1 in goat preadipocyte differentiation remains to be explored.

Adipocyte differentiation is a key step in the process of adipogenesis, and the process through which preadipocytes differentiate into mature adipocytes plays a decisive role for adipogenesis. The differentiation of preadipocytes is a complex biological process which is accompanied by changes of cell morphology and gene expression [16,17], and is regulated by multiple factors and pathways. During the differentiation, the cascade of transcription factors is activated to regulate the expression of adipocyte-development-related genes. PPARγ and C/EBPs are essential transcription factors in adipocyte differentiation [18,19,20], which can activate the expression of differentiation-related genes and promote the lipid accumulation in adipocytes [21,22,23]. Acetyl-CoA carboxylase (ACC) regulates fat synthesis through the adenylate-activated protein kinase (AMPK) pathway [24]. Transcription factors such as SREBP1 and Pref-1 also play an important role in adipocyte differentiation and can be used as marker genes for adipocyte differentiation [25,26]. Lipoprotein lipase (LPL) is a rate-limiting enzyme for the hydrolysis of triglycerides, and its expression level increases gradually during adipocyte differentiation, thus promoting adipocyte differentiation and fat storage [27]. DGAT1 and DGAT2 can catalyze the synthesis of triglycerides, covering almost all triglycerides [28]. Therefore, the expression changes of these marker genes are usually used for indicating adipose precursors’ differentiation. The intracellular lipid accumulation was increased after overexpression of NR4A1 and was decreased after NR4A1 knockdown, which suggested that NR4A1 can promote the differentiation process and the lipid accumulation of adipocytes in goat intramuscular preadipocytes. In order to further determine the effect of NR4A1 on intramuscular preadipocyte differentiation, the changes in the adopigenesis-related marker genes were detected by qPCR. The results showed that overexpression of goat NR4A1 significantly enhanced the expression of *AP2*, *SREBP1*, *ACC*, *GPAM*, and *DGAT2*, indicating that NR4A1 may promote the accumulation of intracellular lipid droplets by up-regulating the expression of ACC and DGAT2, and promote the differentiation of goat intramuscular adipose precursors by up-regulating the expression of AP2, SREBP1, and GPAM. Accordingly, NR4A1 knockdown down-regulated the expression of *LPL*, *C/EBPα*, *C/EBPβ*, *ACC*, and *DGAT2*, thus inhibiting the lipid accumulation of intramuscular adipocytes and the differentiation of goat intramuscular adipocytes. In summary, goat NR4A1 promoted the differentiation of intramuscular preadipocytes by up-regulating the expression of AP2, SREBP1, ACC, GPAM, and DGAT2.

The transcriptome sequencing data of overexpressed goat NR4A1 were analyzed to reveal the underlying mechanisms, and the KEGG enrichment analysis showed that 690 differentially expressed genes were significantly enriched in 86 pathways, including the PI3K-Akt signaling pathway, calcium signaling pathway, Apelin signaling pathway, oxytocin pathway, etc., as well as those related to adipogenic differentiation, such as the AMP signaling pathway [29], IL-17 signaling pathway [30], Wnt signaling pathway [31], Hippo signaling pathway [32], p53 signaling pathway [33], PPAR signaling pathway [34], etc., of which, the PI3K-Akt signaling pathway, cAMP signaling pathway, PPAR signaling pathway, AMPK signaling pathway, Wnt signaling pathway, and other signaling pathways related to adipocyte differentiation and lipid metabolism were selected for further analysis. It was found that there were different degrees of differentially expressed gene enrichment in these pathways; the largest number of genes were enriched in the PI3K-Akt signaling pathway, followed by those enriched in the cAMP signaling pathway, suggesting these signaling pathways interact and regulate each other [35,36]. Studies have shown that the PI3K/Akt signaling pathway can not only increase lipid accumulation [37], but also promote cell adipogenic differentiation [38]. Choe et al. pointed out that water-extracted plum attenuates fat formation in mouse 3T3-L1 adipocytes through the PI3K/Akt signaling pathway [39]. Ohashi et al. [40] found that N1 adenosine oxide has an anti-inflammatory effect through the PI3K/Akt/GSK-3β signaling pathway and promotes osteoblast and adipocyte differentiation. Wang et al. [41] found that phytosterols increase the number of adipocytes and glucose tolerance in mice fed with a high-fat and high-fructose diet by activating the PI3K/Akt signaling pathway. Zhong et al. [42] found that adipose tissue deficient in connective protein LNK has an activated response to IRS1/PI3K/Akt/AS160 signal transduction, while administration of PI3K inhibitors impairs glucose uptake. Based on the transcriptome sequencing results of this study and other studies, we speculate that goat NR4A1 may regulate adipogenic differentiation of intramuscular preadipocytes directly or indirectly through the PI3K-Akt signaling pathway. In order to verify the role of the PI3K-Akt signaling pathway in the promotion of goat intramuscular preadipocytes by NR4A1, LY249002, an inhibitor of the PI3K-Akt signaling pathway, was added after overexpressing NR4A1 in goat intramuscular preadipocytes. The results of oil red O staining showed that the number of lipid droplets in the cells treated with LY249002 decreased significantly, indicating that LY249002 hindered the promoting effect of NR4A1 overexpression on the differentiation of goat intramuscular preadipocytes. At the same time, compared with the control group, the relative expression levels of *C/EBPα*, *AP2*, *SREBP1*, *C/EBPβ*, *ACC*, *GPAM*, *DGAT1*, *DGAT2*, and *ATGL* decreased significantly when LY249002 was added after overexpression of NR4A1, suggesting that LY249002 inhibited the differentiation of goat intramuscular preadipocytes by blocking the overexpression of NR4A1. Taken together, it is suggested that overexpression of NR4A1 may promote the differentiation of goat intramuscular preadipocytes through up-regulating the PI3K-Akt signaling pathway.

## 5. Conclusions

In conclusion, our study confirmed that goat NR4A1 serves as a transcription factor to facilitate the differentiation of intramuscular preadipocytes in goats. NR4A1 overexpression leads to the up-regulation of the expression of *AP2*, *SREBP1*, *ACC*, *GPAM*, and *DGAT* genes, and may, through the PI3K-Akt signaling pathway, assist the adipogenic differentiation. The findings of this study not only identified the potential of NR4A1 as a novel target for goat meat quality improvement and goat molecular breeding, but also provided basic data revealing the molecular mechanisms of the role of NR4A1 in the IMF deposition regulatory network.

## Figures and Tables

**Table 1:** The sequences information of qPCR primers used in this study.

Gene	Primer Sequence (5′~3′)	Tm/°C	Product Length/bp	GenBank Accession Number
*LPL*	S: TCCTGGAGTGACGGAATCTGT	60	174	NM_001285607.1
	A: GACAGCCAGTCCACCACGAT			
*C/EBPα*	S: CCGTGGACAAGAACAGCAAC	58	142	XM_018062278.1
	A: AGGCGGTCATTGTCACTGGT			
*PPARγ*	S: AAGCGTCAGGGTTCCACTATG	60	197	NM_001285658.1
	A: GAACCTGATGGCGTTATGAGAC			
*SREBP*1	S: AAGTGGTGGGCCTCTCTGA	58	127	NM_001285755.1
	A: GCAGGGGTTTCTCGGACT			
*Pref-*1	S: CCGGCTTCATGGATAAGACCT	65	178	KP686197.1
	A: GCCTCGCACTTGTTGAGGAA			
*C/EBPβ*	S: CAAGAAGACGGTGGACAAGC	65	204	XM_018058020.1
	A: AACAAGTTCCGCAGGGTG			
*AP*2	S: TGAAGTCACTCCAGATGACAGG	58	143	NM_001285623.1
	A: TGACACATTCCAGCACCAGC			
*ACC*	S: GGAGACAAACAGGGACCATT	60	146	XM_018064169.1
	A: ATCAGGGACTGCCGAAAC			
*FASN*	S: TGTGCAACTGTGCCCTAG	57	111	NM_001285629.1
	A: GTCCTCTGAGCAGCGTGT			
*ATGL*	S: GGTGCCAATATCATCGAGGT	64	133	NM_001285739.1
	A: CACACCCGTGGCAGTCAG			
*HSL*	S: AGGGTCATTGCCGACTTCC	60	161	XM_018062484.1
	A: GTCTCGTTGCGTTTGTAGTGC			
*GPAM*	S: GCAGGTTTATCCAGTATGGCATT	60	63	XM_013975269.2
	A: GGACTGATATCTTCCTGATCATCTTG			
*DGAT*1	S: CCACTGGGACCTGAGGTGTC	60	111	MT221183.1
	A: GCATCACCACACACCAATTCA			
*DGAT*2	S: CAATAGGTCCAAGGTAGAGAAGC	60	156	NM_001313305.1
	A: ACCAGCCAGGTGAAGTAGAGC			
*UXT*	S: GCAAGTGGATTTGGGCTGTAAC	60	180	XM_005700842.2
	A: ATGGAGTCCTTGGTGAGGTTGT			

S. sense primer; A. antisense primer.

**Table 2:** Primer sequence information used for RNA-seq verification.

Gene	Primers Sequence (5′-3′)	TM/°C	GenBank Accession Number
*RXRG*	S: TCCTCAGGAAAGCACTACGGT	60	XM_005677098.3
	A: GGCAGTATTGACAGCGGTTG		
*FN*3K	S: CGGGAAATGTGGCAGAGGAT	61	XM_018065388.1
	A: TGGTGGTAGGCGGTGAAGAA		
*ANKRD*29	S: GACTCTGCTCCGCCTGCTAC	59	XM_018039771.1
	A: GAGATTGATGTCCGCTCCCT		
*ACSL*1	S: GCCATCACCTACATCATCAACAA	60	XM_005698718.3
	A: ACACTTCTTGCCTCGTTCCA		
*SLC*3A1	S: CACGGTCACTCACTACTCGCA	56	XM_005686551.3
	A: CTGTATCGCCCTGGCTCCCT		
*CMKLR*1	S: ACTACCCCGACGACTTGGAC	62	XM_005691599.3
	A: TCCCGAGGAGGCAGATAATG		
*KLF*2	S: GCGGCAAGACCTACACCAA	60	KU041748.1
	A: TGTGCTTGCGGTAGTGGC		
*CD*36	S: AAAGAACTATTGTGGGGCTA	60	JF690773.1
	A: TATGTGTCAATTATGGCGACT		

S. sense primer; A. antisense primer.

## Data Availability

Data are contained within the article.
